# Supplementary figures and images for: Combinatorial mathematical modelling approaches to interrogate rear retraction dynamics in 3D cell migration
Source: PLoS Comput Biol. 2021 Mar 10;17(3):e1008213. doi: 10.1371/journal.pcbi.1008213 (PMC7984637; doi:10.1371/journal.pcbi.1008213)

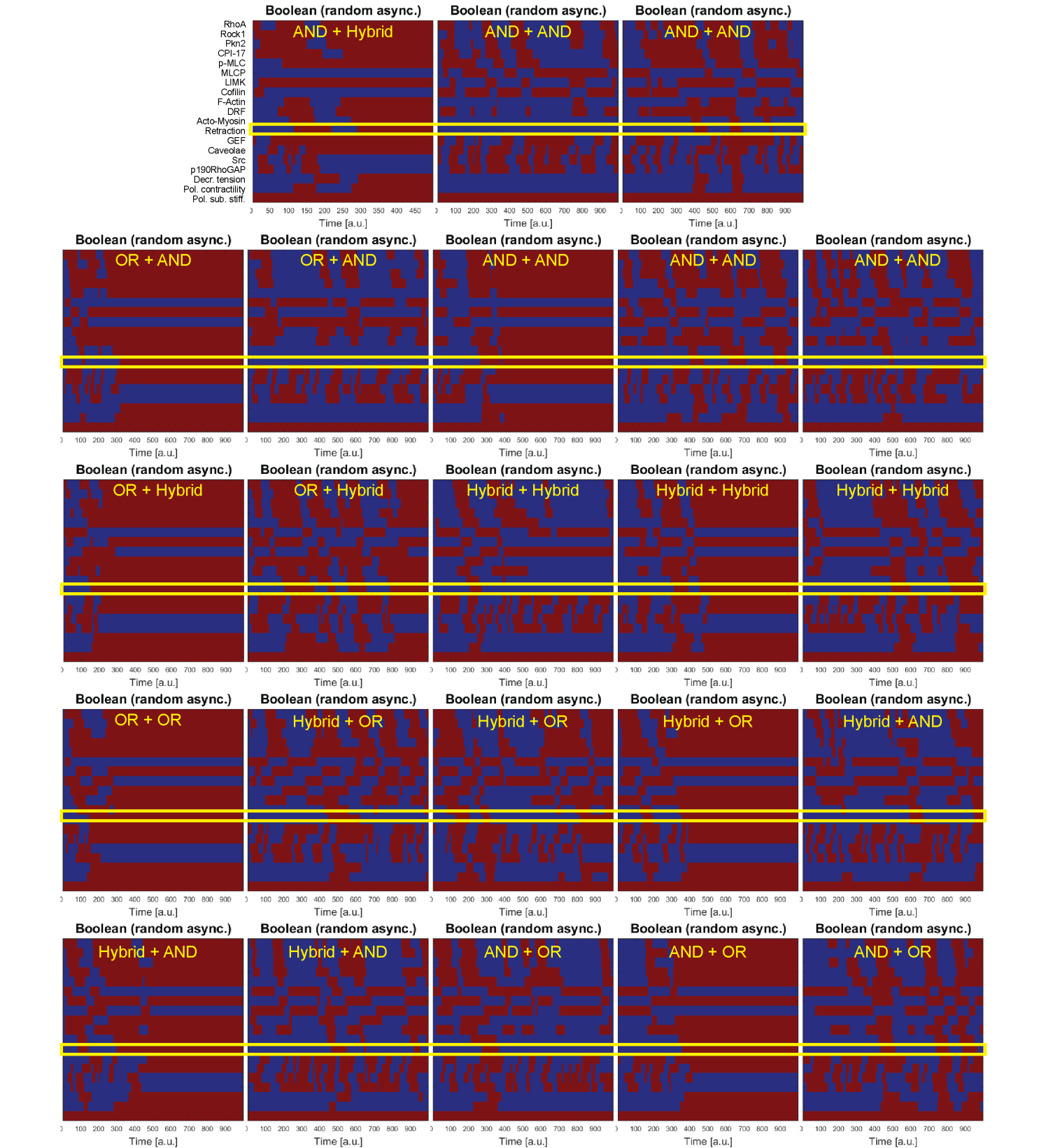

Supplement: S1 Fig — Between the 9 combinations of reaction schemes as in Fig 1B, and the three functional sets of behaviours of rear retraction: steady-state ON, cyclic bursts, steady state OFF, there is a total of 23 corresponding different outputs (i.e. OR + OR scheme only shows steady state rear retraction ON for all 10 stochastic simulations thus only one behaviour is possible whereas the AND + Hybrid scheme exhibits all three permissible behaviours within the 10 simulations). Heatmaps of one of each of these 23 behaviour/reaction scheme combinations are shown with rear retraction highlighted in the yellow box. Outputs were chosen randomly where appropriate. (TIF) [file pcbi.1008213.s001.tif]

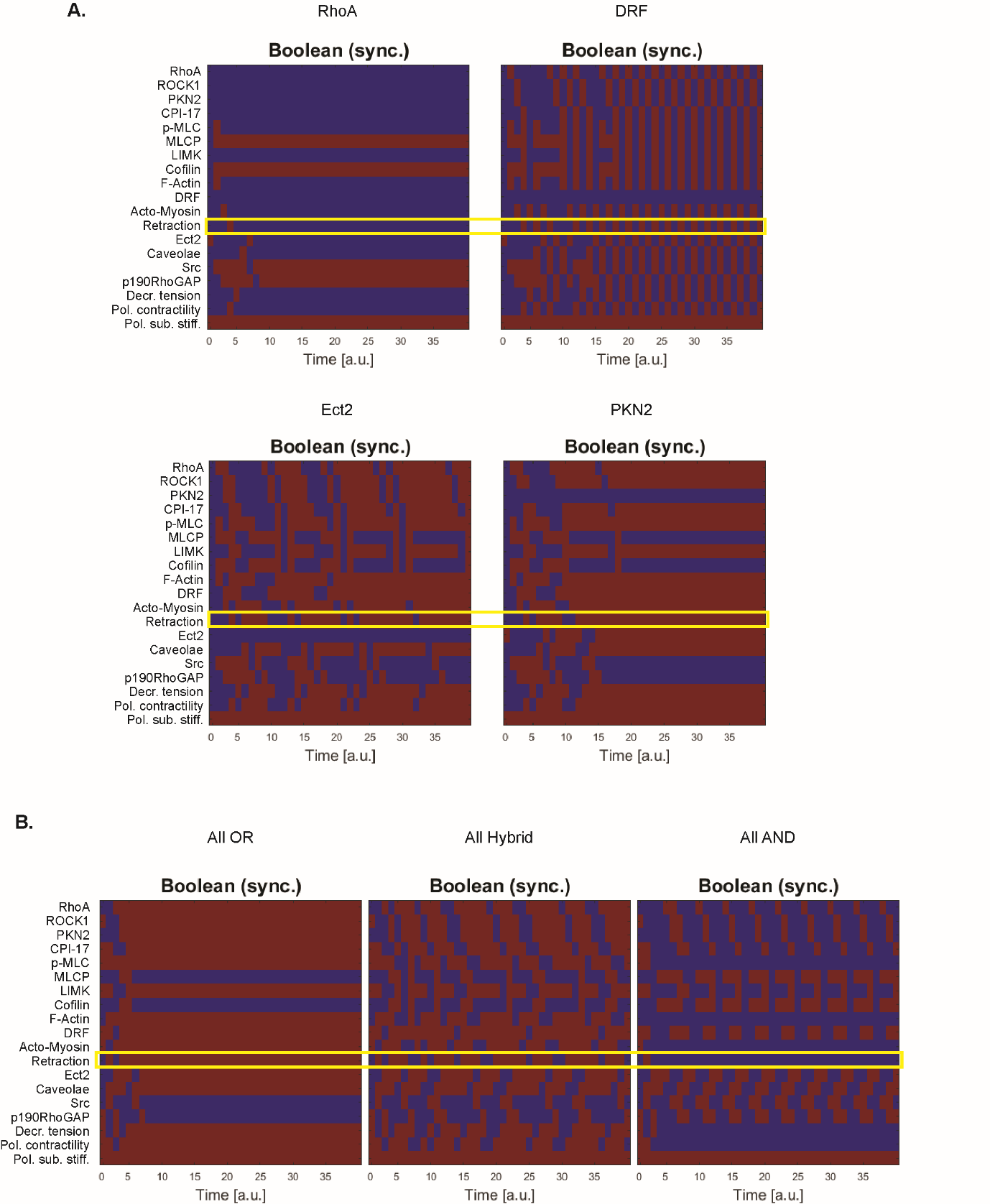

Supplement: S2 Fig — A. Upon node removal (or constitutive activity for inhibitory variables), effects could be grouped into 4 functional sets as summarised in Fig 2D: rear retraction switches OFF, rear retraction switches to persistent oscillatory activity, rear retraction shows cyclic bursts of activity before reaching an ON steady state, and rear retraction remains with an ON steady state. Heatmaps of knockout of RhoA, DRF, GEF and PKN-2 respectively (all in the model without caveolae activation by Src, left in Fig 2D) are shown to exhibit one of each of these behaviours with rear retraction output highlighted in the yellow box. B. Heatmaps of the activity of all variables in the model with the reaction schemes OR + OR + OR (left), Hybrid + Hybrid + Hybrid (centre) and AND + AND + AND (right) given the same set of random initial conditions. Note the set of initial conditions used can be seen by the activity of all the variables at the initial time point (red–ON, blue–OFF). (TIF) [file pcbi.1008213.s002.tif]

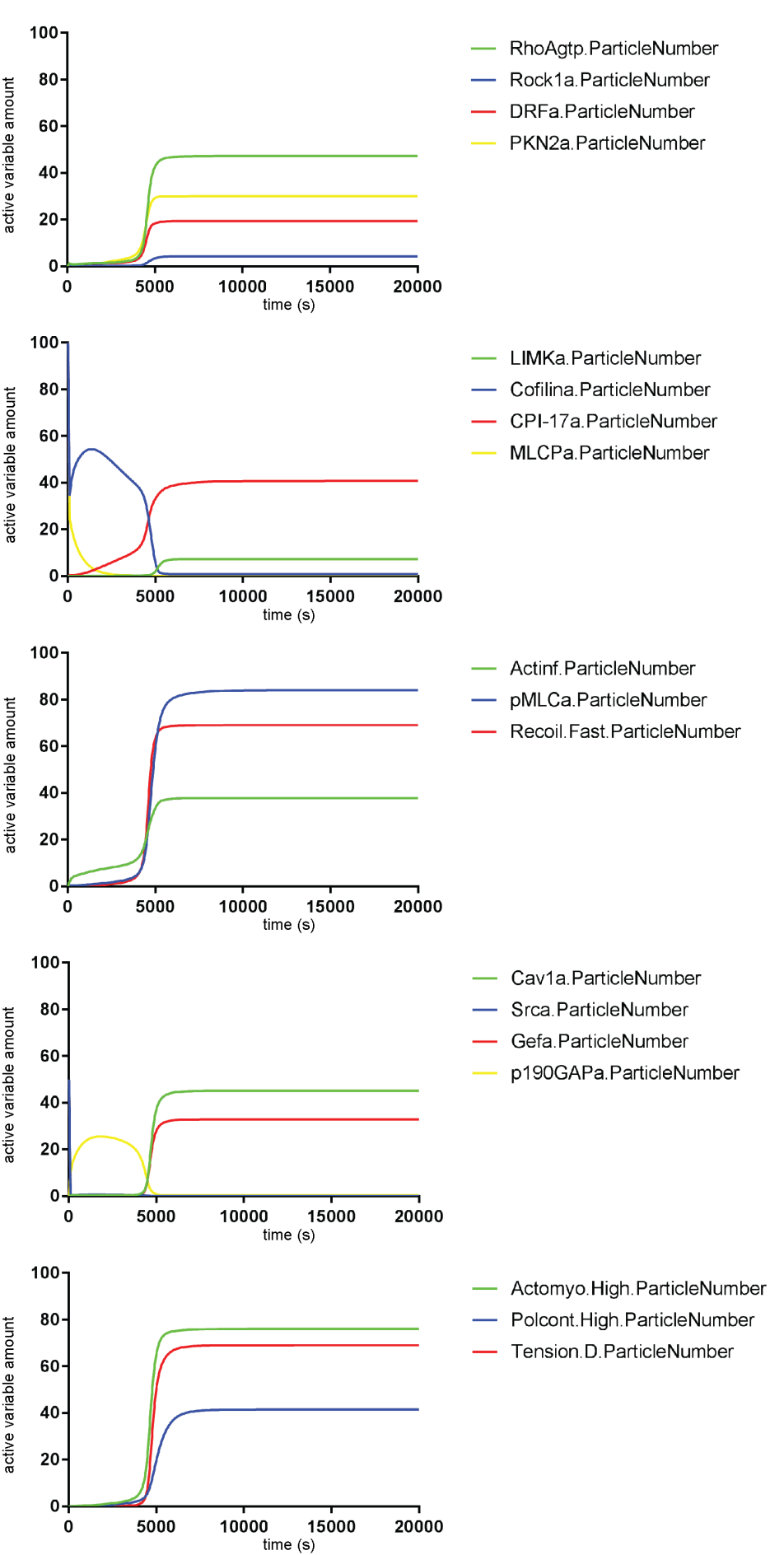

Supplement: S3 Fig — Plots show the activity of the active form of all variables in the model during the first 20,000s. For visual convenience, curves are separated into 5 separate graphs: ranging from RhoA and effectors (top), to biophysical entities (bottom), colours or each curve corresponds to a variable as indicated. Note simulations are all corresponding to the unperturbed simulations in Fig 3A. (TIF) [file pcbi.1008213.s003.tif]

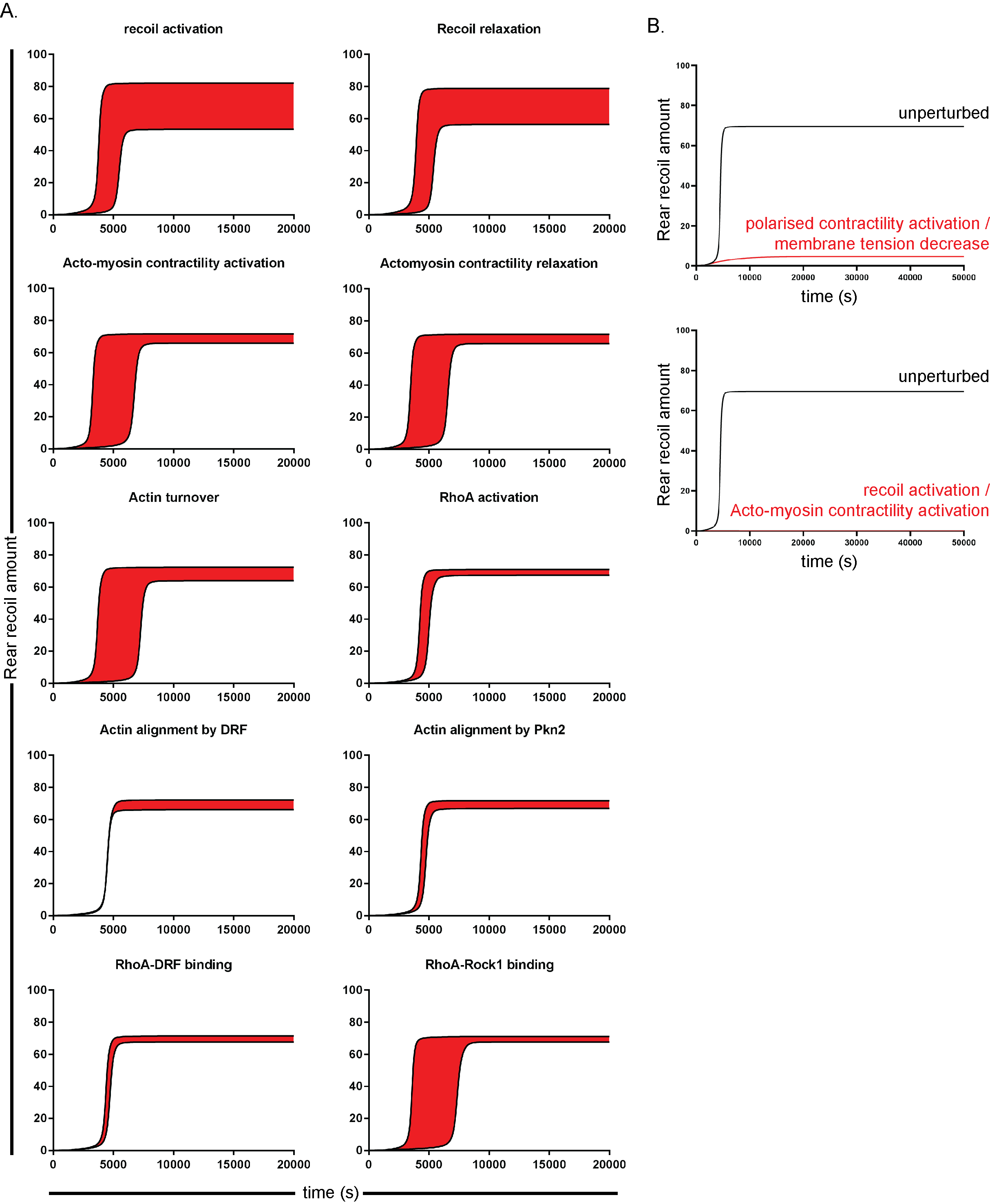

Supplement: S4 Fig — Range of rear retraction temporal dynamics given halving and doubling of the 10 most influential rates in the model. Time course curves of rear recoil amount given halving and doubling of the rate parameters (as shown above each graph) were plotted for the first 20,000 timepoints, then the region in between shaded red on the assumption that this whole region will be covered upon continuous alteration of the rate parameter between these two extremes. B. Deterministic simulation outputs up to t = 50000s given setting each positive regulator reactions with rates less than < 0.0001 to 0. Rates set to 0 shown in red, both rates display the same output dynamics on each graph; curve indistinguishable from x-axis shows that recoil amount = 0 throughout the entire timecourse. (TIF) [file pcbi.1008213.s004.tif]

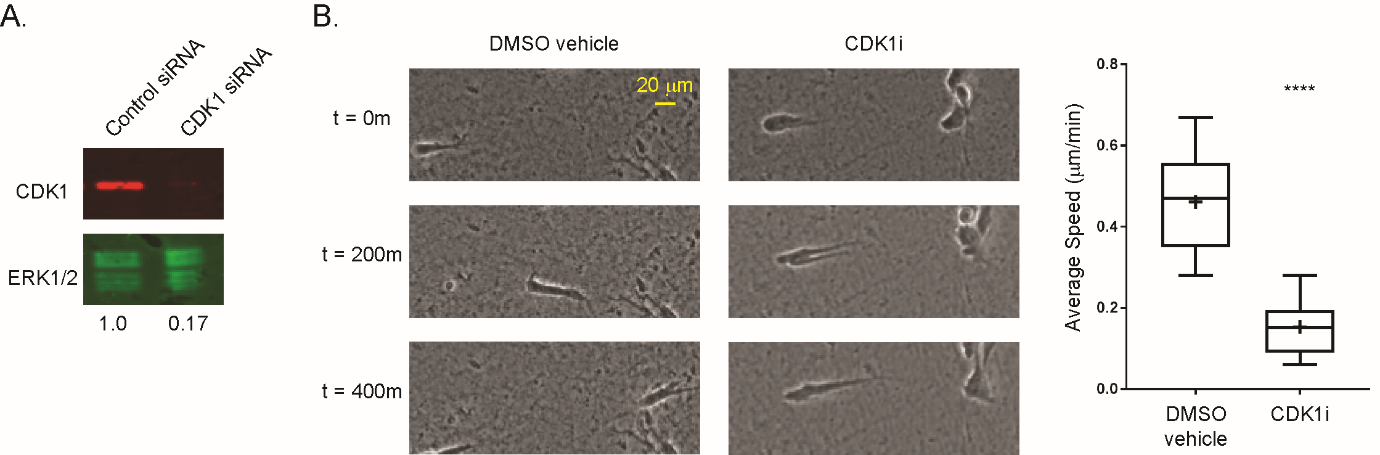

Supplement: S5 Fig — A. Efficiency of knockdown of CDK1 using individual siRNA (band intensity normalised to ERK1/2 loading shown below blots). B. Left: A2780 cells were treated with DMSO (left) or CDK1 inhibitor (right) and seeded in CDM and imaged by high-end widefield microscopy across 16 hours, representative individual cells shown across t = 400 minutes, Right: Quantification of average speed of control or CDK1 inhibited cells during 16h timecourse, (N = 30 cells across 2 repeats analysed per condition). (TIF) [file pcbi.1008213.s005.tif]

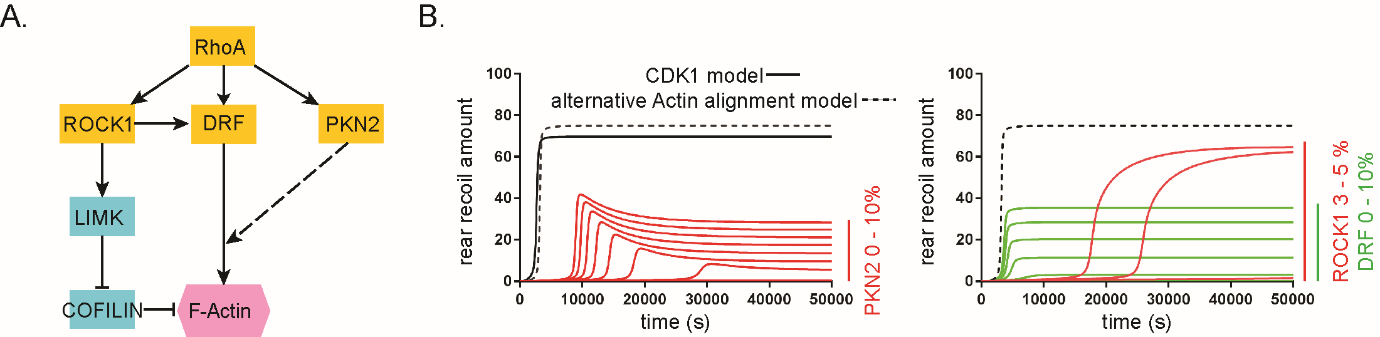

Supplement: S6 Fig — A. Alternative formulation of model concerning RhoA signalling to aligned actin where F-actin alignment now requires coordinated activity of both DRF and PKN-2 together. B. Timecourse plots of rear retraction amounts in response to in silico knockdown/reductions in levels of PKN-2 between 0 and 10% (left), ROCK1 between 3 and 5% (red lines, right) or DRF between 0 and 10% (green lines, right). (TIF) [file pcbi.1008213.s006.tif]

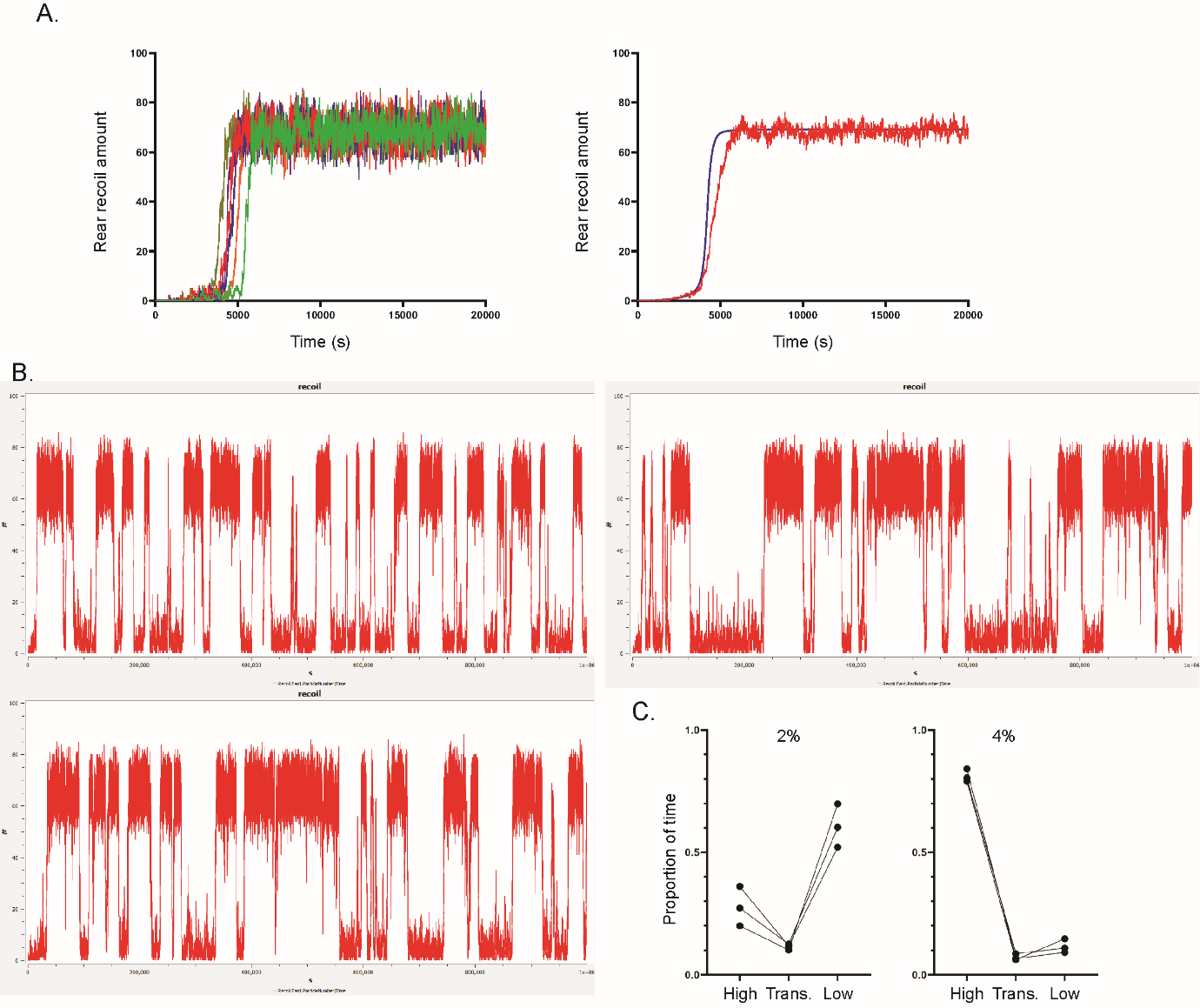

Supplement: S7 Fig — A. 6 stochastic simulations showing rear recoil amount with respect to time for the first 20,000s when run with exactly the same reactions and parameters as in the CDK1-included ODE model in Fig 5 (left) and the average of the 6 individual stochastic simulations (red line) in comparison with the previous deterministic curve (blue line) for rear retraction amount with the same parameters (right). B. Timecourse plots of the original model of rear recoil during stochastic simulations for the first 1,000,000s with polarised substrate stiffness set to 4%. C. Proportion of time of the 1,000,000s timecourses rear recoil amount spent in the high state (defined as rear recoil > 50%), the low state (< 20%) or transitioning (trans.) between (>20%, <50%) of 3 simulations with substrate stiffness polarity set at 2% or 4% with the CDK1 model. (TIF) [file pcbi.1008213.s007.tif]

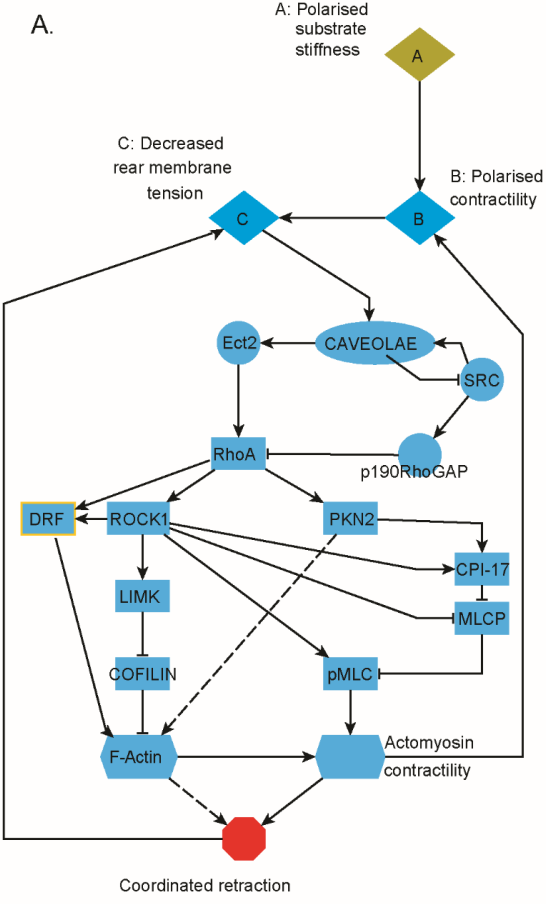

Supplement: S1 Boolean model — Compatible with CellNetAnalyzer (a MatLab plug-in), see methods. (ZIP) [file pcbi.1008213.s012.zip › Hetmanski Boolean rear model 2020/boolean network-1.bmp]
